# The Babies

**Published:** 1888-07

**Authors:** 


### THE BABIES.

The great aim of the physicians is the preservation of human life, and how we shall manage the babies, the great question of the day. As the warm weather of summer approaches, we all begin to feel in anticipation our sympathies aroused for these little embryo men and women—the pride of the father's eye and the darling of the mother's heart.

The question of dress is a very important one, and one that always causes the thinking mother, who devotes herself less to fashion than to her responsibilities to her household, to pass many anxious moments. But perhaps the most important question of all in the management of the child is its proper feeding. How many little graves are made, how many happy homes and loving hearts are made desolate because of the loss of little ones that are yearly sacrificed to improper feeding? It behooves us, then, as men, to whom the health of the home is entrusted, to give our time and best thought to the plans best suited to nourishing the little members of the household.

All properly enlightened people are a unit as to the fact that *human* milk is of all things *the* food for the infant. Then the first question arises, is there any means by which we may cause the mother's health and strength to hold up and the breasts, that God gave her to nourish her offspring to more perfectly perform their functions? For answer let us state a case. Having attended a lady in her confinement in the spring, the writer was, during the early summer months, consulted by her as to the proper substance and manner of preparation of a suitable food for the baby. The mother exhibited that peculiar nervous, broken-down appearance so often seen when women have passed sleepless and anxious nights caused by the fretting of an ill-fed or insufficiently nourished child. Said she, "I am giving very little milk and my baby is slowly starving—see its sunken eyes, flabby skin, its emaciated legs. It does not seem sick, and yet I know it is slowly dying and that too, of hunger." My reply was, "Have you a cow in full milk?" "No." "Then," said I, "send your husband out and have him buy the best cow he can find, put her in the best pasture, feed her well, see that she is properly and thoroughly milked." "Doctor, do you think then her milk will agree with my baby?" "Yes, after *you* have drunk the milk and digested it, you will give as much good, pure milk as your child can drink, and it will most certainly agree with both you and your baby." The result was, the mother who had from her girlhood been of rather frail, delicate constitution, and, as her family supposed, inclined to consumption, became rounded and plump, her breasts soon began to respond, and she gave quantities of nutritious milk, more than her baby could consume, and I heard no more of any artificial baby-food. I have tried this plan repeatedly and have never known it to fail. Try it. If your patient is one of those unfortunate persons who does not like sweet milk, educate her, and if she is a mother in the true sense of the word she will be an apt scholar. Do not advise too much milk at a time, but let her drink it regularly and increase the quantity as the stomach becomes more tolerant. She will soon learn to take a glass of cold milk when thirsty instead of water. Do you want the reason the prescription? Then I quote from Flint's Human Physiology: "It is a curious fact, which has been fully established by observation upon the human subject and the inferior animals, that, while the quantity of milk is increased by taking a large amount of simple water, *the solid constituents are also increased, and the milk retains all its qualities as a nutritive fluid.* (*Italics mine.*)

But suppose, for reasons beyond our control, we cannot have the mother nurse the child, or a wet nurse of proper kind to take her place, then what shall we do? In the first place we must stop and consider the indications of nature. It is reasonable to suppose that the digestive tract only gets into condition to digest properly *pari passu* with the ability of the organs of mastication to suitably prepare the food to be digested by these organs. Hence, we would not expect the stomach to digest any substances that would require chewing until nature has given the proper organs of mastication—the teeth. Nature does not indicate; rational man should not expect it. I hold that no child should ever be fed anything but milk until it shall have gotten its full set of teeth. To prepare these foods so as not to require mastication and to enable the child to swallow them without choking, simply transfers the dangers from the organs of deglutition to those of digestion, and only delays the evil hour; for while in the one case quick and certain death ensues—in the other slow torture and equally certain death ends the scene.

A comparison of the composition of human and cows milk shows the human milk to be poorer in nitrogenized principles and richer in butter and sugar. This should be our guide. Milk should never be fed to a child that has been long drawn, or that has been hauled in a wagon. Take, say three or four quarts—or three times the quantity you will need for twelve hours—of *freshly drawn* cow's milk from a healthy cow or healthy cows and place it in a perfectly clean vessel to stand for an hour at a temperature of sixty degrees—pour off one-third of the top of this milk —dilute it with a proper quantity of water according to age or strength of the child, gradually bring it to the boiling point, add a little lime water, or a small quantity of pure bicarb soda will do; put in a little sugar and you have the best substitute for the mother's milk ever prepared. Place it in a clean well stopped bottle and put it on ice or in very cold water—feed warm in small quantities from a common prescription vial with a rubber nipple. The reason why the milk should be put to stand before pouring off "the baby's milk" is this ; the portions of milk containing the caseine or curdy portion—which is by far the most important of the nitrogenized principles of the milk and of which we do not want so much as the cow's milk contains—tends toward the bottom, while the buttery portions, of which we want more in proportion than we find in the cow's milk, tends toward the top. We add the alkali because the mother's milk is always alkaline and we frequently find cow's milk of acid reaction; and besides it tends to prevent coagulation of the caseine. We add the sugar because we find the cow's milk a little less rich in saccharine matter. Prepared and fed as stated, we have no large undigested curds to form in the stomach to irritate and inflame the digestive tract, and we have a food, that while it gives a sufficient plumpness to the child, also develops bone and sinew and muscle. It entails wonderful trouble, but it gives trouble with a healthy, growing, crowing, winsome baby ; mother and child sleep soundly and sweetly at night, and the druggist is no more disturbed to dispense his carminatives and soothing syrups. Put it down in your case book among poisons and their antidotes that the much vaunted and widely spread infant's foods now on the market are not what we want for our little patients, but pure, freshly drawn, properly prepared and properly fed cow's milk.

If you want a substitute for fresh cow's milk, the next thing best to be used is the dessicated or condensed milk ; but this like all substitutes for substitutes gets us further away from the source and adds new dangers as well as greater troubles. The rearing of children is a serious and troublesome problem and the most important factor is the feeding. No woman has the right to marry and risk bringing into the world a child, unless she is willing to devote her time and attention to its proper care; and what we want is a food which is wholesome and digestible and at the same time one that will not require the powers of an expert chemist to properly prepare it. To the *mothers* should be entrusted the care and preparation of the infant's food; and we agree with Woodbury that women capable of making such chemical experiments as would be required in the preparation of a great many of the so-called infant's food, are either teaching in some college, or, if married, rarely if ever have babies. "C."

